# Assessing SARS-CoV-2 Neutralizing Antibodies after BNT162b2 Vaccination and Their Correlation with SARS-CoV-2 IgG Anti-S1, Anti-RBD and Anti-S2 Serological Titers

**DOI:** 10.3390/diagnostics12010205

**Published:** 2022-01-15

**Authors:** Angélica Ramos, Maria João Cardoso, Luís Ribeiro, João Tiago Guimarães

**Affiliations:** 1 Serviço de Patologia Clínica, Centro Hospitalar e Universitário de São João, 4200-319 Porto, Portugal; princesamjc@gmail.com (M.J.C.); luis.ribeiro@chsj.min-saude.pt (L.R.); jtguimar@med.up.pt (J.T.G.); 2 EPI Unit, Instituto de Saúde Pública, Universidade do Porto, 4200-135 Porto, Portugal; 3 Departamento de Biomedicina, Faculdade de Medicina, Universidade do Porto, 4200-135 Porto, Portugal

**Keywords:** SARS-CoV-2, neutralizing antibodies, surrogate virus neutralization test, serological immunoassays, BNT162b2 vaccine

## Abstract

The humoral response through neutralizing antibodies (NAbs) is a key component of the immune response to COVID-19. However, the plaque reduction neutralization test (PRNT), the gold standard for determining NAbs, is technically demanding, time-consuming and requires BSL-3 conditions. Correlating the NAbs and total antibodies levels, assessed by generalized and automated serological tests, is crucial. Through a commercial surrogate virus neutralization test (sVNT), we aimed to evaluate the production of SARS-CoV-2 NAbs in a set of vaccinated healthcare workers and to correlate these NAbs with the SARS-CoV-2 IgG anti-S1, anti-RBD and anti-S2 serological titers. We found that 6 months after vaccination, only 74% maintain NAbs for the Wuhan strain/UK variant (V1) and 47% maintain NAbs for the South African and Brazil variants (V2). Through Spearman’s correlation, we found the following correlations between the percentage of inhibition of NAbs and the SARS-CoV-2 IgG II Quant (Abbott Laboratories, Chicago, IL, USA) and BioPlex 2200 SARS-CoV-2 IgG Panel (Bio-Rad, Hercules, CA, USA) immunoassays: rho = 0.87 (V1) and rho = 0.73 (V2) for anti-S1 assessed by Abbott assay; rho = 0.77 (V1) and rho = 0.72 (V2) for anti-S1, rho = 0.88 (V1) and rho = 0.82 (V2) for anti-RBD, and rho = 0.68 (V1) and rho = 0.60 (V2) for anti-S2 assessed by BioPlex assay (*p* < 0.001 for all). In conclusion, we found a strong correlation between this fast, user-friendly, mobile and bio-safe sVNT and the serological immunoassays.

## 1. Introduction

Clinical trial data indicate that the mRNA vaccines are approximately 95% effective at preventing COVID-19 [1,2]. Additionally, several real-world studies after vaccine administration have confirmed their effectiveness in controlling the COVID-19 pandemic [3,4,5,6]. However, the durability of this protection remains unknown, and the influence of new variants still needs to be studied. The humoral immune response, which occurs mainly through the production of neutralizing antibodies (NAbs), is a key component of the protective immune response to COVID-19 [7,8,9]. Thus, the assessment of NAbs against SARS-CoV-2 has a critical role in monitoring the effectiveness and durability of vaccine-induced immunity to follow the efficacy of vaccination.

Since the beginning of the COVID-19 pandemic, several commercial SARS-CoV-2 serologic tests have been developed and widely used as complementary diagnostic tools, for seroprevalence studies and, later, for the evaluation of vaccine performance [10,11,12,13]. However, these tests determine the total binding antibodies, and it is known that only a small subset of these antibodies are capable of neutralizing viruses and protecting against future infection and disease [14,15]. 

The possibility to study this neutralizing capacity is rather cumbersome and shows several limitations. Indeed, the plaque reduction neutralization test (PRNT), considered the gold standard for measuring NAb levels, has several limitations: it is technically demanding, needs to be performed in high biosecurity laboratories (BSL-3), has very low throughput and has a long turnaround time [16,17,18,19]. Thus, it is necessary to develop and validate alternative methods to assess NAb production. 

The surrogate virus neutralization test (sVNT) detects NAbs without the need to manipulate live viruses or cells and can be completed in 1–2 h in a BSL-2 laboratory. Using purified receptor-binding domain (RBD) from the Spike protein (S) and the host cell receptor ACE2, the test is designed to mimic the SARS-CoV-2-host interaction in an ELISA or a fluorescent immunoassay (FIA). Beyond this, in a clinical laboratory, where serological tests are highly generalized and automated, it is also crucial to correlate the serological titer with the antibodies’ neutralization capacity.

Therefore, using a commercial sVNT, our aim was to evaluate SARS-CoV-2 NAb production in a set of vaccinated healthcare workers after 6 months (BNT162b2) and correlate it with the SARS-CoV-2 IgG anti-S1, anti-RBD and anti-S2 serological titers. In a small subgroup of participants, we also assessed the production of NAbs immediately before and 1 month after the 3rd dose inoculation.

## 2. Materials and Methods

Six months after full vaccination, we invited 89 healthcare workers from Centro Hospitalar Universitário de São João (CHUSJ) to participate in a neutralizing antibody study. We selected these participants from a group that we have been monitoring for the production of SARS-CoV-2 IgG since the day after the start of the vaccination process.

This study population seemed interesting to us for two reasons. First, we observed an abrupt decrease in total antibody production 6 months after the inoculation of two doses of Pfizer vaccine, but we knew nothing about the production of NAbs. Second, the variability of serological titers observed would allow us to study the relationship between total and neutralizing antibody levels.

Thus, participants were selected according to anti-S1 serological titer, previously determined by the SARS-CoV-2 IgG II Quant assay (Abbott Laboratories, Chicago, IL, USA). In this way, they were distributed into the following 3 serological subgroups: 37 with a titer < 820 AU/mL, 16 with a titer between 1300–1600 AU/mL and 36 with a titer > 2110 AU/mL. 

The anti-S1, anti-RBD and anti-S2 titers were also determined in 55 participants by the BioPlex 2200 SARS-CoV-2 IgG Panel (Bio-Rad, Hercules, CA, USA) and correlated with NAb levels. Regarding the RBD, 15 participants were excluded because, despite an additional dilution, the values remained outside (above) the measuring range of the immunoassay. 

In a subgroup of NAb-positive healthcare workers at 6 months after vaccination (n = 21), we investigated what happened to NAbs immediately before inoculation of the 3rd dose, which in our hospital occurred 10 months after the 2nd shot. In 13 of these participants, we also assessed the production of NAbs at 1 month after the 3rd inoculation.

In a subgroup of NAb-negative participants at the 6th month mark, NAbs were also evaluated at 3 months after the 2nd dose (n = 8) and 1 month after the 3rd dose (n = 13).

Among the 89 healthcare workers, 5 are men and 84 are women, with mean ages of 47 (IQR 38–62) and 47 (IQR 22–63), respectively. Participants completed an epidemiological survey, where they did not report prior COVID-19. Furthermore, through the BioPlex 2200 SARS-CoV-2 IgG Panel, all participants at 1, 3 and 6 months after vaccination were negative for the production of IgG anti-N, which confirms the absence of previous SARS-CoV-2 infection.

The serological assays were performed according to the manufacturer’s instructions. Their selection was based on the availability of the corresponding automatic analysers in our laboratory and the experience of many months with these two immunoassays in particular, whether in the performance of seroprevalence studies or in the assessment of humoral immunity in COVID-19 patients and immunocompromised subgroups, as well as in vaccinated populations. 

### Standard F SARS-CoV-2 nAb FIA

NAbs were accessed through the Standard F SARS-CoV-2 nAb FIA (SD Biosensor, Cheongwon-gun, Korea), a sVNT for the qualitative measurement of circulating NAbs against 2 groups of SARS-CoV-2 variants in human serum and plasma. Variant Group 1 (V1) includes the Wuhan strain and UK variant (B.1.1.7); Variant Group 2 (V2) includes the South African variant (B.1.351) and Brazil variant (P.1). 

The test principle is as follows: Anti-RBD antibody and streptavidin coat the region of the control line and the region of the test lines, respectively, on the surface of a nitrocellulose membrane. During testing, the RBD-conjugated protein with europium particles interacts with SARS-CoV-2 NAbs in the sample, forming the antibody-RBD complex, or with the ACE-2 protein conjugated with biotin, forming the ACE2-RBD complex. The ACE2-biotin and RBD-europium complex migrates along the membrane by capillary action until reaching the test line, where it is captured by the streptavidin. The fluorescence light appears in inverse proportion to the NAbs amount. 

The test was carried out according to the manufacturer’s instructions. Briefly, 200 µL of buffer was added to 2 microtubes previously labeled with V1 and V2; 100 µL of the specimen was added and vortex; lyophilized beads of RBD and ACE-2 were dissolved in the mixture inside the microtubes; the mixture was incubated at 37 ± 1 °C for 20 min; 100 µL of the mixture was applied to the test device and incubated for 15 min; and fluorescence was read on the STANDARD F200 analyzer (SD Biosensor).

The results are given in percentage of inhibition (PI) according to the formula:


$$\;\mathrm{PI}\left(\%\right)\;=\;\left[1-\left(\frac{\mathrm{Specimen}\;\mathrm{fluoroscense}\;\mathrm{intensity}}{\mathrm{Aver}.\;\mathrm{Negative}\;\mathrm{fluoroscence}\;\mathrm{intensity}\;\left(\mathrm{factor}\;\mathrm{calibration}\right)}\right)\right]\times 100$$

A PI < 20% is reported as “negative,” and a PI ≥ 20% is reported as “positive.”

The correlation between the PI and serological titer against the different immunogenic proteins and the different immunoassays was calculated through Spearman’s correlation.

The study was approved by the CHUSJ ethics committee.

## 3. Results

The SARS-CoV-2 NAbs for V1 and V2 were observed in 74% and 47% of the healthcare workers, respectively, with a mean PI of 55% (V1) and 43% (V2). The percentage of participants with NAbs and the mean PI distributed across the 3 serological subgroups is shown in Table 1. 

Through Spearman’s correlation, we found a rho = 0.87 (V1) and rho = 0.73 (V2) between PI and SARS-CoV-2 IgG anti-S1 titer accessed by Abbott immunoassay (*p* < 0.001 for all). When we correlated the NAb levels with SARS-CoV-2 IgG titers from BioPlex, we found a rho = 0.77 (V1) and rho = 0.72 (V2) between PI and anti-S1 titers, a rho = 0.88 (V1) and rho = 0.82 (V2) between PI and anti-RBD titers and a rho = 0.68 (V1) and rho = 0.60 (V2) between PI and anti-S2 titers (*p* < 0.001 for all).

**Figure 1:**
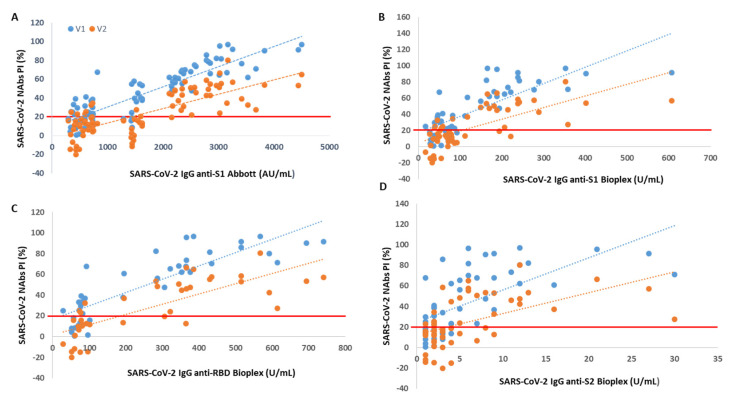
Percentage of SARS-CoV-2 neutralizing inhibition (PI) as a function of SARS-CoV-2 IgG serological titer. The PI of NAbs for V1 (blue) and V2 (orange) are correlated with (**A**) anti-S1 (Abbott), (**B**) anti-S1 (BioPlex), (**C**) anti-RBD and (**D**) anti-S2 serological titer. The red line represents the sVNT cut-off. PI results < 20% are reported as “negative,” and PI ≥ 20% are reported as “positive”.

We found that from an anti-S1 titer ≥ 2110 AU/mL (Abbott), anti-S1 titer ≥ 112 U/mL (BioPlex), anti-RBD titer ≥ 193 U/mL and anti-S2 titer ≥ 5 U/mL, all participants showed circulating NAbs for V1, with mean PIs of 72%, 74%, 74% and 70%, respectively. From an anti-S1 titer ≥ 2158 AU/mL (Abbott), anti-S1 titer ≥ 238 U/mL (BioPlex), anti-RBD titer ≥ 366 U/mL and anti-S2 titer ≥ 11 U/mL, all participants produced NAbs for V2, with mean PI’s of 46%, 50%, 57% and 51%, respectively. In Figure 1, we show PI as a function of SARS-CoV-2 IgG titers. 

**Figure 2:**
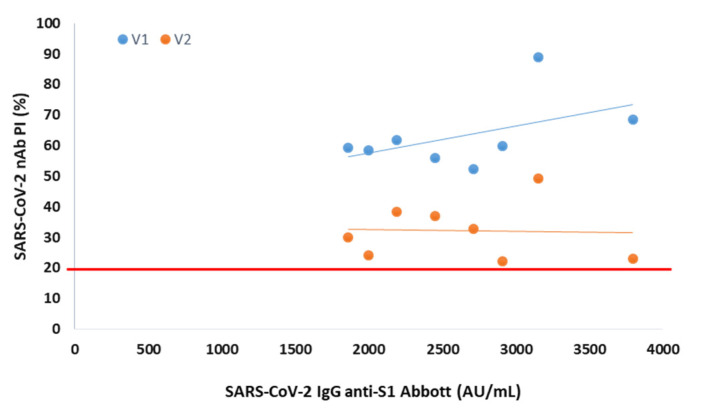
Percentage of SARS-CoV-2 neutralizing inhibition (PI) as a function of SARS-CoV-2 IgG anti-S1 in serum collected 3 months after vaccination from NAbs-negative participants. The PI of NAbs for V1 (blue) and V2 (orange) are correlated with anti-S1 (Abbott) serological titer in 8 NAbs-negative participants at 6 months after vaccination. The red line represents the sVNT cut-off. PI results < 20% are reported as “negative,” and PI ≥ 20% are reported as “positive”.

In serum collected 3 months after vaccination from 8 participants negative at the 6th month, the presence of NAbs was observed for both variants, with mean PIs of 63% (V1) and 32% (V2) (Figure 2). 

**Figure 3:**
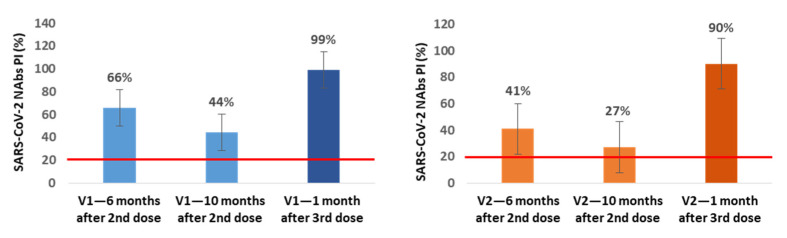
Mean percentages of SARS-CoV-2 neutralizing inhibition (PI) at 6 and 10 months (n = 21) after the 2nd dose and 1 month (n = 13) after the 3rd dose of Pfizer vaccine for V1 (blue) and V2 (orange). The red line represents the sVNT cut-off. PI results < 20% are reported as “negative,” and PI ≥ 20% are reported as “positive”.

In a small subgroup of participants, we also assessed the production of NAbs immediately before and 1 month after the 3rd dose inoculation. In the subgroup of NAbs-positive healthcare workers at 6 months after vaccination (n = 21), we found an additional average decrease of 22% (V1) and 14% (V2) in NAbs PI at 10 months after the 2nd dose, corresponding to the time when our healthcare workers were vaccinated with the 3rd dose. In 13 of these participants, we also assessed the production of NAbs at the 1st month after the 3rd inoculation, and we observed an average increase of 55% (V1) and 63% (V2) in NAbs PI compared with the values for the 10th month (Figure 3). 

In all of the NAbs-negative participants at the 6-month mark in which the NAbs were evaluated at 1 month after the 3rd dose (n = 13), we found NAbs production for V1 and V2 with average PI values of 99.8% for both variants. 

## 4. Discussion

Through a sVNT, we determined the SARS-CoV-2 NAbs in a group of vaccinated healthcare workers without previous COVID-19. We found that 6 months after vaccination, only 74% maintain NAbs for the Wuhan strain and for the UK variant. This percentage drops to 47% when we look at the South African and Brazil variants. 

The decline in total and neutralizing antibodies 6 months after vaccination has been described [20,21,22,23]. Likewise, the decrease in the effectiveness of vaccines associated with SARS-CoV-2 variants has also been reported [24,25,26].

In December 2020, a rapidly growing lineage in the UK associated with an unexpectedly large number of genetic changes was reported [27]. Within 1 month, two additional lineages, with a significant number of mutations, were identified from South Africa and Brazil [28,29]. The difference in vaccine efficacy for the different variants is easily explained since the vaccine was developed for the Wuhan strain. As the new mutations give rise to variants whose spike protein is less homologous to the original strain, the ability of its RBD domain to bind the ACE-2 receptor on human cells and thus neutralize the virus may decrease, as our data showed, in line with other studies [24,25,26]. In one of them, published in The Lancet (June 2021), Emma C Wall and colleagues reported a decrease of 5.8, 4.9 and 2.6-fold in NAbs levels for the Delta, South Africa and UK variants, respectively, in relation to the Wuhan strain [24].

After the UK, South Africa and Brazil variants, a new variant, the Delta variant (B.1.617.2), emerged in India, quickly increasing its prevalence from 2% in February 2021 to 87% in May 2021 [30]. Since then, the Delta variant has spread widely in multiple countries and displayed evidence of being even more transmissible than the UK variant and causing more severe disease than earlier variants [30]. In November 2021, the Omicron variant (B.1.1.529) was identified in South Africa and, since then, has caused super spreader events and outcompeted Delta within weeks in several countries [31]. Furthermore, an unprecedented number of mutations in its spike gene may result in reduced vaccine effectiveness. Therefore, it would have been very important to determine the SARS-CoV-2 NAbs for the Delta and Omicron variants; this is a limitation of this study. In an updated version of this commercial immunoassay, it is crucial to include these variants [32,33,34].

Another finding of this work is that the presence of NAbs, as well as the percentage of inhibition, is directly proportional to the SARS-CoV-2 IgG anti-S1, anti-RBD and anti-S2 serological titer. This is truly relevant as serological tests have a far more widespread use than neutralization tests, are easier to carry out, faster, more standardized and easier to scale to perform studies at the populational level. Thus, it is essential to be able to establish a relationship between the serological titer and the neutralization capacity. Here, we found a strong correlation between the NAbs PI from Standard F SARS-CoV-2 nAb FIA and the SARS-CoV-2 IgG II Quant and BioPlex 2200 SARS-CoV-2 IgG Panel immunoassays. The highest correlation was found for SARS-CoV-2 antibodies against RBD, followed by those against S1. We also determined the following minimum neutralizing serological titers for both variants: 2158 AU/mL, 238 U/mL, 366 U/mL and 11 U/mL for anti-S1 (Abbott), anti-S1 (BioPlex), anti-RBD and anti-S2 proteins, respectively. This strong correlation and the possibility of establishing a neutralizing serological cut-off were also described by other authors [35,36].

In 8 participants without NAbs for V1 and V2 at 6 months after vaccination, we observed that they were positive for NAbs at 3 months after vaccination. These results ensure that these individuals are capable of producing NAbs, excluding any biological peculiarities. Additionally, they once again reinforce the link between the decline of total and neutralizing antibodies over time.

From the subgroup of participants in which we assessed the production of NAbs immediately before and at 1 month after inoculation with the 3rd dose, we concluded that between the 6th and 10th month, NAbs continued to decrease, reaching mean PI values of 44% (V1) and 27% (V2), and that the 3rd booster dose increases these levels to mean values of 99% (V1) and 90% (V2), even in participants who were NAbs-negative at the 6th month after the 2nd dose; these individuals achieved mean PI values of 99.8% for both variants.

We believe that this real-world study provides us with important data concerning the duration and effectiveness of vaccine-induced protection. However, it has several limitations: the number of participants is small; COVID-19 patients were not evaluated; only one type of vaccine was included; and the NAbs determination method is not the gold standard. It would be important in future studies for this fast, user-friendly, mobile and bio-safe sVNT to be evaluated using the PRNT as a reference method. Furthermore, we have to highlight that when we study total or neutralizing antibodies, we are not including cellular immunity, which can be maintained even after antibodies decay.

## Figures and Tables

**Table 1:** Percentage of participants with NAbs and the mean PIs distributed across the 3 serological subgroups assessed by SARS-CoV-2 IgG II Quant assay.

Serological Titer (U/mL)	NAbs V1	NAbs V2	% PI V1	% PI V2
<820	19/37 (51%)	6/37 (16%)	32	15
1300–1600	11/16 (69%)	2/16 (13%)	44	13
>2110	36/36 (100%)	34/36 (95%)	72	46

## Data Availability

Not applicable.
